# Pulmonary Manifestations of IBD: Case Report and Review of the Literature

**DOI:** 10.3390/jcm13185401

**Published:** 2024-09-12

**Authors:** Amit Herling, Tal Moshe Perluk, Ophir Freund, Nitsan Maharshak, Nathaniel Aviv Cohen

**Affiliations:** 1 Faculty of Medicine, Ben-Gurion University of the Negev, Be′er Sheva 8410501, Israel; amith9254@gmail.com; 2 Faculty of Medical and Health Sciences, Tel Aviv University, Tel Aviv 6139001, Israel; talmp@tlvmc.gov.il (T.M.P.); ophir068@gmail.com (O.F.); nitsanm@tlvmc.gov.il (N.M.); 3 The Pulmonary Institute, Tel Aviv Medical Center, Tel Aviv 6423906, Israel; 4 IBD Unit, Department of Gastroenterology and Liver Diseases, Tel Aviv Medical Center, Tel Aviv 6423906, Israel

**Keywords:** inflammatory bowel disease, Crohn’s disease, ulcerative colitis, pulmonary manifestations, case report

## Abstract

This article explores the pulmonary complications associated with inflammatory bowel disease (IBD). It presents a detailed case study of a 22-year-old male with Crohn’s disease exhibiting pulmonary symptoms. The review delves into the spectrum of pulmonary involvement in IBD, covering clinical presentations, diagnostic challenges, underlying pathophysiology, and management strategies. It highlights the significance of these extraintestinal manifestations on patient outcomes and quality of life. The article underscores the need for heightened clinical awareness and a systematic approach to diagnosis and management, integrating the expertise of multiple specialists. The review identifies gaps in current research, suggesting avenues for future investigation to enhance the understanding and treatment of these complex manifestations.

## 1. Case Report

A 22-year-old male, non-smoker, with no significant medical history presented with persistent symptoms including diarrhea and vomiting for approximately six weeks. The symptoms were accompanied by abdominal pain intense enough to disturb his sleep, with a trend of worsening over time. At presentation he had 4–6 watery bowel movements a day, occasionally accompanied by blood. Additionally, during the two years prior to presentation, he reported a chronic cough, at times producing scant amounts of blood-stained mucus, and bilateral knee pain over the last three months. No family history of auto-immune conditions or inflammatory bowel diseases (IBD) was noted.

Initial diagnostic investigations included upper endoscopy which revealed extensive ulceration involving the antrum, stomach body, and proximal portion of the duodenum. Colonoscopy identified multiple ulcers extending from the descending colon to the cecum with aphthous ulcers and small ulcerations in the terminal ileum. Histopathology confirmed the diagnosis of Crohn’s Disease (CD). According to the Montreal Classification he was classified as A2, B1, L3, with L4 modifier for upper gastrointestinal CD.

**Figure 1:**
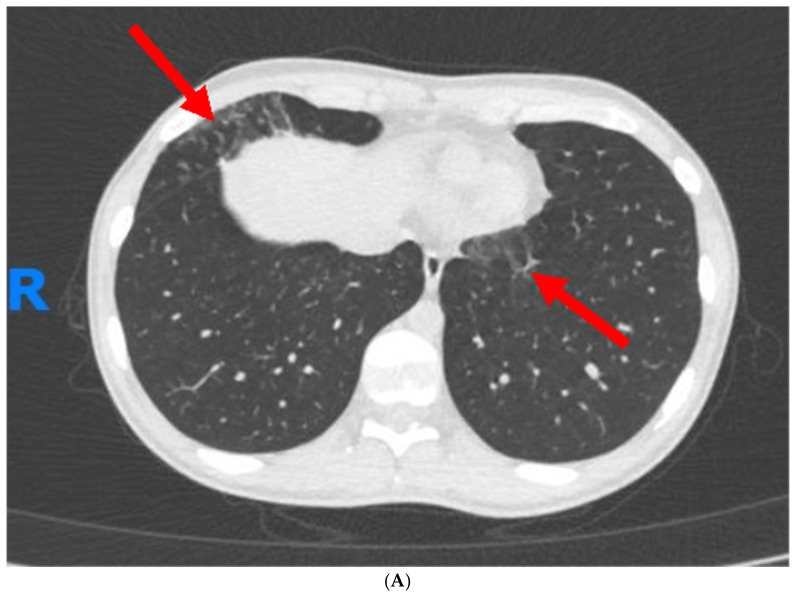

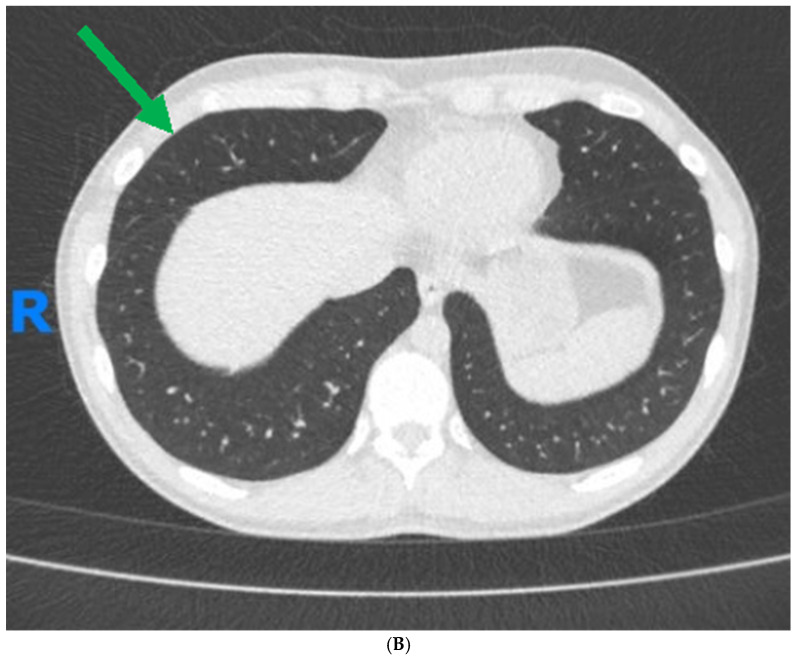
(**A**)—Chest CT scan revealed subtle opacities in a tree-in-bud pattern in the lower and right middle lobes of the lungs (red arrows), along with minute, non-specific nodules in the lingula and upper lobes. (**B**)—Follow-up chest CT scan, four months post-treatment initiation, indicated complete resolution of the previously noted nodular opacities (green arrow) and other abnormal findings.

A computed tomography enterography (CTE) was performed to determine disease extent and showed inflammation in the terminal ileum, right colon, and cecum, with lesser involvement of the transverse colon. An additional finding was observed in the small bronchi at the lung bases. A chest computed tomography (CT) scan was subsequently performed to better define these changes and it revealed subtle opacities in a tree-in-bud pattern in the lower and right middle lobes of the lungs, along with minute, non-specific nodules in the lingula and upper lobes (Figure 1A). No evidence of bronchiectasis, interstitial disease, or enlarged hilar or mediastinal lymph nodes were observed. These findings were consistent across two chest CT scans conducted three weeks apart, with no significant changes noted between them. The differential diagnosis for these pulmonary changes included chronic inflammatory changes in small airways, extraintestinal involvement of CD, micro-aspirations, or ones secondary to a viral/atypical infection.

The patient was followed-up by a specialist pulmonologist and had further testing performed including spirometry, plethysmography, and diffusing capacity of the lung for carbon monoxide which were all within normal limits. Methacholine challenge was also negative. Tuberculosis testing was negative. As the patient’s symptoms were chronic, his travel and exposure history were negative, and no fever was present—it was felt that this was unlikely to be of infectious origin.

Following multidisciplinary consultation with an IBD expert, pulmonologist, and radiologist, it was decided to start therapy for his CD. The patient commenced treatment with infliximab at a standard dosing of 5 mg/kg at weeks 0, 2, and 6, followed by bimonthly administration thereafter in combination with 6-mercaptopurine at a dose of 1 mg/kg daily. He initially received oral prednisone at a dose of 40 mg tapered over 2 months.

At follow-up, four months post-treatment initiation, the patient was in corticosteroid-free remission from a CD perspective and reported resolution of his cough. Concurrently, a follow-up chest CT scan indicated complete resolution of the previously noted nodular opacities and other abnormal findings (Figure 1B).

## 2. Introduction

Inflammatory bowel diseases (IBD), Crohn’s disease (CD), and ulcerative colitis (UC) are characterized by chronic inflammation primarily affecting the gastrointestinal tract, leading to symptoms and complications which can severely impact a patient’s quality of life [1]. Patients with IBD often suffer from extra-intestinal manifestations (EIM) involving multiple organ systems, and these highlight the systemic impact of IBD beyond the digestive system [2].

One of the less commonly encountered, or at least reported upon EIMs in IBD, are those involving the pulmonary system. Small case-control studies, case reports, and epidemiological population-based studies have contributed to our understanding of these manifestations [3], however, the prevalence of pulmonary manifestations in IBD is not well defined due to variability in study designs and diagnostic criteria [4]. It appears that the prevalence of these manifestations varies across different populations and regions, and they can occur in both adult and pediatric IBD patients [5]. Of interest is the finding that the onset of pulmonary symptoms has been noted to coincide with post-operative periods, especially after colectomy, suggesting a possible link with surgical intervention [6,7]. A study by Desai et al. (2011) showed that the prevalence of pulmonary manifestations in IBD patients in India is significant, with approximately 28.5% of IBD patients exhibiting abnormal pulmonary function tests. Abnormal HRCT findings were present in 22% of patients, including bronchiectasis, nodules, and other abnormalities. It is important to note that most patients with these abnormalities were asymptomatic [8]. Higher rates of obstructive respiratory diseases, such as asthma (8.6%) and COPD (8.7%), are observed in adult IBD patients compared to the general population. Additionally, there is an increased prevalence of bronchiectasis (46% higher), pulmonary vasculitis (52% higher), and interstitial pneumonia (52% higher) in IBD patients compared to controls [9].

Pulmonary manifestations associated with IBD represent a spectrum of disorders, the manifestations of which can occur at any stage of the gastrointestinal disease and may present with a variety of clinical symptoms, ranging from subclinical to overt pulmonary disease [10,11]. The pulmonary manifestations of IBD are diverse and can range from airway disease to lung parenchymal disease, thromboembolic disease, pleural disease, enteric-pulmonary fistulas, pulmonary function test abnormalities, and adverse drug reactions [12].

The differential diagnosis for pulmonary manifestations linked with IBD encompasses a range of respiratory disorders. These may present either as comorbidities secondary to the primary IBD pathology or as distinct primary respiratory diseases. The relationship between IBD and other chronic diseases, particularly those affecting the respiratory system, is an area of clinical interest due to the implications it has for patient care and understanding the systemic nature of IBD [13].

In this review we detail the various aspects of pulmonary involvement in patients with IBD, and aim to provide future areas of research to help expand our understanding of this EIM in patients with IBD.

## 3. Clinical Presentation of Pulmonary Involvement in Patients with IBD

The clinical presentation of pulmonary manifestations in patients with IBD can be diverse, ranging from asymptomatic cases detected incidentally through imaging to severe respiratory distress requiring urgent care. The onset of these manifestations may precede, coincide with, or follow the diagnosis of IBD, and they can fluctuate independently of the intestinal disease activity. The most frequently reported symptoms include chronic cough, dyspnea, and chest discomfort, which are not necessarily correlated with the activity of the bowel disease [14,15].

These symptoms are not specific to IBD-related pulmonary disease and can often be mistaken for more common respiratory conditions. Some patients may also exhibit acute respiratory symptoms that resemble pneumonia but do not respond to conventional antibiotic therapy, leading to further investigation that reveals underlying IBD-related pathology.

The clinical manifestation of the pulmonary involvement can be categorized based on the respiratory tract involvement. Airway involvement can present as large airway pathology, such as tracheobronchitis, or small airway disease manifesting as bronchiolitis. Bronchiectasis characterized by permanent dilation of bronchi due to chronic inflammation and infection is a well-documented manifestation in patients with IBD. Patients with bronchiectasis often present with symptoms like a productive cough and recurrent chest infections [16,17,18].

Interstitial lung diseases (ILDs) are also associated with IBD and can range from transient to chronic conditions, and usually present as progressive dyspnea, often accompanied by a productive cough [19]. ILD is more associated with UC, while granulomatous lung disease has been associated with CD [20].

The serosal surface of the lung can also be involved. This serositis can present as pleuritic chest pain and pleural effusions. Cryptogenic organizing pneumonia (COP) has been frequently reported in IBD patients [17]. The disease often presents with non-specific symptoms, including malaise, cough, fever, dyspnea, and a restrictive pattern in pulmonary function tests, and bronchoalveolar lavage frequently reveals lymphocytosis [21]. The impact of pulmonary involvement on the quality of life of patients with IBD can be substantial. Respiratory symptoms may significantly impair physical activity and may also complicate the management of the underlying bowel disease.

## 4. Pathophysiology of Pulmonary Involvement in IBD

The pathophysiological mechanisms underpinning the pulmonary manifestations of IBD are complex and have not been fully elucidated. However, several hypotheses and findings from clinical studies provide insight into potential links between the respiratory and gastrointestinal systems in the context of IBD.

### 4.1. Immune-Mediated Inflammation

Several theories suggest that IBD impacts systemic inflammation which can subsequently involve the lungs through a disruption in the body’s immune response. In patients with IBD, the usually precise migration system of lymphocytes, guided by chemokines, becomes indiscriminate. Such a transformation can lead to lymphocytes erroneously targeting organs like the lungs, in addition to the gut.

The gut–lung axis plays a crucial role in this pathogenesis, as the chronic intestinal inflammation in IBD compromises the intestinal barrier, allowing antigens to translocate. These antigens activate immune cells, including dendritic cells and macrophages, which then release cytokines, such as interleukin (IL)-1, IL-2, IL-6, tumor necrosis (TNF)-α, and interferon-gamma [22]. These substances have the potential to affect various processes of the body, including cell adhesion, white blood cell movement, and the creation of harmful oxidative compounds, which might contribute to damage in the lung tissue [23] and could explain the variety of organ systems involved in IBD [12,24].

Furthermore, there is an embryologic connection between the lungs and colon, both originating from the early foregut and having similar structural features such as columnar epithelia [25]. Their shared mucosal immune system can respond similarly to antigens introduced through inhalation and ingestion, possibly triggering inflammation in both areas. Additionally, activated memory T-cells from the gut can migrate to lung tissues, demonstrating a cross-talk between the gut and lungs, thus further establishing the concept of the gut–lung axis in IBD [22].

Another important factor is that patients with IBD have an increased risk of developing other autoimmune and inflammatory diseases such as primary sclerosing cholangitis (PSC), celiac disease, type 1 diabetes, rheumatoid arthritis, and ankylosing spondylitis [13]. Amongst these conditions are also immune-mediated diseases that affect the pulmonary system, like sarcoidosis and asthma.

### 4.2. Genetic Predisposition

Genetic factors may predispose individuals to both IBD and pulmonary manifestations. Certain genetic markers are associated with IBD and pulmonary disease like asthma and COPD, indicating a possible genetic link. Moreover, a population-based retrospective study demonstrated that the incidence of IBD was significantly higher among patients with asthma and COPD compared to the general population of Québec, Canada, further strengthening this theory [4].

Asthma has been the most extensively studied comorbidity in the context of IBD. Epidemiological studies have identified an increased prevalence of asthma in patients with IBD, and conversely, IBD is more common among patients with asthma compared to the general population. The underlying mechanisms for this association may involve shared genetic susceptibility, environmental triggers, and common inflammatory pathways [26].

Gasdermin-B (GSDMB) is a protein crucial for epithelial cell development and differentiation.

A study that investigated the impact of gene polymorphisms on GSDMB structure found that specific single nucleotide polymorphisms (SNPs) impact GSDMB’s regulatory activity and the ability to bind sulfatide, a lipid involved in maintaining epithelial cell integrity and implicated in inflammation. This binding is linked to various disease mechanisms, for instance in UC, sulfatide interaction with natural-killer (NK) T-cells can lead to tissue damage. Moreover, GSDMB overexpression has been connected to asthmatic symptoms in mice, suggesting a role in pulmonary manifestations and a link between gut and pulmonary inflammation [27].

There also appears to be a genetic connection between COPD and IBD. Patients with COPD are at a higher risk of developing IBD, and this risk increases with the severity of COPD, with smoking playing a key role in both diseases, exacerbating CD while potentially protecting against UC [28,29,30,31]. The connection is primarily suggested by the overlap in certain genetic factors, nucleotide-binding oligomerization domain containing 2 (NOD2), and the hedgehog-interacting protein (HHIP) gene [25]. Mutations in gene coding for NOD2 has been observed in populations with COPD. This mutation is already known to significantly increase the risk for CD and CD-related complications. In addition, HHIP, which is associated with COPD, is also important in the development of the intestinal crypt axis. As such, the presence of these mutations in both COPD and IBD patient populations suggests a potential genetic bridge between pulmonary and gut inflammation. Further studies are needed to explore the extent and nature of this relationship.

### 4.3. Microbial Translocation and the Gut–Lung Axis

The gut–lung axis and microbial translocation can contribute to the pathophysiology of pulmonary involvement in IBD. Dysbiosis, a disruption in the gut’s microbial balance, often triggered by environmental factors such as diet, antibiotics, and stress, can lead to a reduction in beneficial bacterial species and an increase in pathogenic ones. This imbalance in the gut microbiota facilitates microbial translocation, where microbes and their metabolites escape the gut and enter systemic circulation. Such translocation disrupts both tissue and immune homeostasis, linking gut health directly to lung health. In the context of the gut–lung axis, this microbial translocation can exacerbate pulmonary conditions, increasing susceptibility to airway diseases and infections [17,31].

### 4.4. Environmental Factors

Environmental factors, including smoking and air pollution, have been implicated in both the exacerbation of IBD and the development of lung disease. These factors may act as triggers for the onset of pulmonary manifestations in susceptible individuals [4,31,32].

The relationship between smoking and IBD, specifically CD, as well as its connection to pulmonary disease, has been extensively studied, revealing complex interactions.

Smoking is positively associated with CD, with smokers having a significantly higher risk of developing CD compared to non-smokers. The risk increases with the number of cigarettes smoked per day and the duration of the habit [33]. This finding is corroborated by a meta-analysis that confirmed smoking as an important environmental factor for CD, with differing effects observed in UC [34]. Smoking not only increases the risk for CD but also affects its clinical course, necessitating more aggressive treatment approaches. Smokers with CD are more likely to require glucocorticoids and immunosuppressive drugs, indicating a more severe disease course [35]. Furthermore, smoking is linked with a higher incidence of surgery in patients with CD, suggesting its contribution to disease refractoriness. This association emphasizes the need for smoking cessation as a part of comprehensive disease management [36].

Chronic cigarette smoke exposure has been shown to induce systemic hypoxia that drives intestinal dysfunction. This is evidenced by the development of CD-associated pathology in the colon and ileum, including gut mucosal tissue hypoxia and increased risk and severity of CD in a mouse model of smoke-induced experimental COPD [37]. A cross-sectional cohort study demonstrated that smoking is associated with extra-intestinal manifestations in patients with IBD [38].

### 4.5. Drug-Induced Pulmonary Toxicity

Medication used in the treatment of IBD, such as sulfasalazine, mesalamine, and methotrexate, can have pulmonary side effects (Table 1) [16]. Recognizing and understanding these drug-induced pulmonary effects is crucial for clinicians to balance the benefits of IBD treatment against potential respiratory risks [39]. The pathophysiology of drug-induced pulmonary injury may involve direct toxic effects on the pulmonary epithelium, hypersensitivity reactions, or indirect effects such as drug-induced systemic lupus erythematosus [40]. These mechanisms can lead to a wide range of pulmonary pathologies, including interstitial lung disease, eosinophilic pneumonia, and organizing pneumonia. The diagnosis of drug-induced pulmonary complications can be challenging, often requiring a combination of clinical, radiographic, and pathological findings. Immunosuppression from IBD-related drugs could also lead to pulmonary complications in a non-direct mechanism, including repeated pulmonary infections and bronchiectasis [41].

### 4.6. Vascular Involvement

Studies indicate a notable correlation between IBD and various forms of vasculitis, suggesting a higher frequency of vasculitis in patients with IBD than previously thought. This relationship is particularly evident in large-vessel vasculitis (LVV), ANCA-associated vasculitis (AAV), and granulomatous vasculitis—affecting the skin and mesenteric arteries, which have been observed in some patients with CD. It appears that a diagnosis of IBD often precedes the onset of vasculitis, and most of the patients affected by both are women [51,52]. Another condition observed in patients with IBD is IgA vasculitis (IgAV), with studies implicating anti-TNF-α therapies in its emergence [53,54].

The literature illustrates a heightened risk of thromboembolic events in patients with encompassing deep vein thrombosis (DVT) and pulmonary embolism (PE), with estimates suggesting a three-fold increase in risk compared to controls [55]. This association is underscored by findings that patients experience these events at younger ages relative to the general population [56,57]. The pathogenesis of thromboembolic complications in IBD is multifactorial, involving a mix of genetic, immune-mediated, and inflammatory factors that collectively foster a prothrombotic state [58,59]. Given the serious implications of thromboembolic disease in this patient population, including increased morbidity and mortality, a high degree of vigilance and proactive management strategies are essential for early detection and intervention.

### 4.7. Enteric-Pulmonary Fistulas

Enteric-pulmonary fistulas represent a rare yet significant complication in patients with IBD, particularly CD. The prevalence of fistulas in CD varies, but they are a common complication. The cumulative incidence of any fistula has been estimated to be 33% after 10 years and 50% after 20 years after diagnosis, specifically perianal fistulas [60]. However, enteric-pulmonary fistulas, such as colobronchial, ileobronchial, and esophagobronchial fistulas, are less commonly reported, with most information coming from single case studies [12,61,62,63]. These fistulas usually extend from various sections of the gastrointestinal tract to different parts of the lung, often following the path of least resistance and anatomical proximity. For example, colobronchial fistulas typically stretch from the colon’s splenic flexure to the lower lobe of the left lung, attributed to the closeness of these structures [12].

Fecopneumothorax is an extremely rare and potentially life-threatening complication, which involves the presence of air and fecal matter within the pleural cavity, typically resulting from a perforation in the gastrointestinal tract into the thoracic cavity. This condition can be iatrogenic as a complication of esophagectomy or colonic perforation in a diaphragmatic hernia [64,65,66].

The diagnosis of these fistulas is based on a thorough clinical examination coupled with specific diagnostic procedures. Indicators such as recurrent pneumonia and feculent sputum should prompt the consideration of an enteric-pulmonary fistula. Diagnostic confirmation can be achieved through abdominal and thoracic CT scans, MRI and an enema using a water-soluble contrast medium, with providing further details on the disease’s extent and the presence of any abscesses or fluid collections [67]. Such fistulas, particularly colopleural fistula and fecopneumothorax, are rare but pose life-threatening risks, necessitating immediate surgical intervention upon diagnosis.

## 5. Available Diagnostic Modalities and the Challenges Associated with Diagnosis of Pulmonary EIMs

Diagnostic evaluation of IBD-related pulmonary symptoms involves a multifaceted approach [68]. Diagnosing pulmonary manifestations in IBD is challenging due to the broad differential diagnosis that includes common conditions like asthma, COPD, infections, and drug-induced lung disease [16]. Moreover, the lack of awareness of pulmonary IBD manifestations can lead to under diagnosis or misdiagnosis, requiring high index of suspicion, especially in patients with known IBD presenting with new or worsening respiratory symptoms.

A thorough assessment for additional etiologies or factors associated with respiratory diseases is crucial, including exposures, medications and family history. Structured questionnaires can be used for this purpose, like the CHEST ILD questionnaire, which was shown to add valuable information that was not identified in the office visit [69].

Despite these approaches, current diagnostic methods have significant limitations. The clinical overlap between IBD-related pulmonary manifestations and other respiratory conditions can make accurate diagnosis challenging. Imaging and pulmonary function tests may not always effectively diagnose IBD-related respiratory symptoms, as asymptomatic patients can have abnormal findings on HRCT and PFT, potentially leading to diagnostic errors [70,71]. Additionally, the lack of standardized protocols for assessing pulmonary involvement in IBD can result in variability in diagnosis and management across different healthcare settings.

Additional evaluation of suspected pulmonary involvement in IBD typically includes a combination of pulmonary function tests (PFTs), imaging [72], and sometimes invasive procedures (Table 2). These challenges highlight the need for improved diagnostic tools that are more sensitive and specific for detecting pulmonary manifestations in IBD. Future research should aim to develop and validate such tools to enhance early and accurate diagnosis, which is critical for timely and effective treatment.

Collectively, these diagnostic tools not only facilitate the detection of pulmonary involvement in IBD but also help differentiate IBD-related pulmonary disease from other etiologies, including drug-induced conditions and concomitant respiratory diseases like asthma.

## 6. Management of Pulmonary Manifestations in Patients with IBD

The management and treatment of pulmonary manifestations in patients with IBD require a multidisciplinary approach that takes into consideration the complexity of both the intestinal and respiratory conditions. When pulmonary symptoms are identified in patients with IBD, a careful evaluation is necessary to determine if these are directly related to IBD, medication side effects, or a separate pulmonary condition. The management strategy is then tailored based on the underlying etiology, severity of symptoms, and the patient’s overall clinical status.

### 6.1. Pharmacological Treatment Options

The literature on pharmacological treatments for IBD-related pulmonary manifestations mainly relies on case reports and expert opinion [76,77]. Cases of spontaneous resolution have been reported, suggesting that delayed diagnosis could lead to overlooked cases. Corticosteroids are the fundamental treatment in roughly two-thirds of cases, but up to one-third of patients either do not respond to this treatment or fail to undergo tapering off [78]. Infliximab, an anti-tumor necrosis factor (TNF) agent, shows promise as an effective alternative or adjunctive therapy, particularly in cases where corticosteroids are ineffective [79].

Corticosteroids: These are frequently the initial treatment for inflammatory pulmonary complications associated with IBD [24]. They can be administered orally or intravenously, depending on the severity of the condition, whereas a combination of inhaled and oral corticosteroids at high doses is recommended, depending on the nature of pulmonary involvement [39,78,80]. Corticosteroids are effective in most cases of IBD-related respiratory disorders, including bronchiectasis, drug-induced pulmonary toxicity caused by sulfasalazine, mesalamine, and methotrexate [12,75,81]. Notably, corticosteroid failure is more common in cases involving the small airways where alternative therapies should be sought [80].

Biological Therapies: The introduction of biological therapies into the IBD therapy armamentarium over the last two decades has dramatically changed the therapeutic landscape. Many of these therapies, for example anti-TNF agents, have shown efficacy in treating other EIMs of IBD [82]. Regarding pulmonary EIMs, these therapies have been reported to be effective at alleviating obstructive abnormalities in pulmonary function tests observed in patients with active IBD, however it remains unclear if the improvement in pulmonary function tests (PFTs) is directly due to the anti-inflammatory effects of anti-TNF at the pulmonary level or if it is a result of improved gastrointestinal inflammation due to the therapy [70]. It must be kept in mind that anti-TNF agents do increase the risk of reactivation of tuberculosis, as well as pneumonia, and, as such, these conditions need to be ruled out and vaccinated against, respectively, before initiating these therapies [83].

Antibiotics: Antibiotics are prescribed in cases of secondary infection, particularly in bronchiectasis associated with IBD [3,12,84]. The main antibiotics used are macrolides due to their anti-inflammatory properties. These properties have been well documented in the literature related to non-cystic fibrosis bronchiectasis, where azithromycin significantly reduced the exacerbation rate, although it did not lead to detectable improvements in functional outcomes or quality of life [77,85]. Importantly, non-tuberculosis mycobacteria infection must be ruled-out prior to macrolide imitation to prevent the development of macrolide-resistance.

Immunosuppressants: In the management of ILDs associated with systemic sclerosis (SSc), cyclophosphamide and mycophenolate mofetil have emerged as a viable treatment option [86], possibly reflecting broader applicability to autoimmune-related pulmonary manifestations, including those linked to IBD. Cyclophosphamide, particularly when administered intravenously and followed by maintenance therapy with drugs such as azathioprine, can stabilize or improve pulmonary function in a significant proportion of patients with systemic autoimmune conditions [87,88]. While cyclophosphamide is effective, mycophenolate mofetil (MMF) might offer a better safety profile, suggesting a preference for MMF in managing SSc-ILD, despite cyclophosphamide’s proven efficacy. Despite its benefits, cyclophosphamide’s use requires careful patient selection and monitoring due to potential adverse effects, including infection risks and bone marrow suppression [89].

### 6.2. Non-Pharmacologic Interventions

Pulmonary Rehabilitation may be beneficial for patients with chronic respiratory symptoms, helping to improve lung function and quality of life [90]. Although evidence of its effectiveness varies between lung diseases, the high safety and positive overall outcomes show promise in less common diseases, such as IBD-related lung disease. Patients with advanced pulmonary disease and hypoxemia may require supplemental oxygen, although its effects on long-term outcomes should be validated.

The benefits of vaccination for patients with IBD are substantial, especially given the increased risk of infectious diseases associated with both the condition itself and the immunosuppressive therapies often required for treatment [91,92]. Vaccinations offer protection against a wide range of infections, including influenza and pneumococcal pneumonia [93]. The immunosuppressive drugs used in IBD management increase susceptibility to infectious diseases. Vaccination provides an added layer of protection for these patients, helping to safeguard them from potential infections that could lead to hospitalizations or more severe complications [94,95]. Furthermore, by preventing infections, vaccinations allow for the continued use of necessary immunosuppressive therapies without the interruptions that might occur due to infection-related complications. This is crucial for maintaining the effectiveness of IBD treatment regimens.

Surgery is rarely suggested for IBD-related pulmonary manifestations, but may be considered in severe cases of bronchiectasis or when there is significant lung damage that does not respond to medical therapy or enteropulmonary fistulae [11].

While uncommon, there have been case reports where IBD-related lung parenchymal manifestations have resolved without modifying or stopping the pharmacological treatment [96].

The treatment of IBD-related pulmonary manifestations is multi-modal and requires collaboration between the IBD specialists, pulmonologist, radiologist, and other personnel based on specific cases. The cornerstone lies in controlling inflammation, and the choice of therapy depends on the specific pulmonary condition, its severity, and other comorbidities. Regular follow-up with pulmonary function tests and imaging studies is important to monitor disease progression and treatment response. In cases where pulmonary symptoms are attributed to medications used for IBD, discontinuation of the implicated drug, when feasible, often results in the resolution of symptoms. When the drug cannot be stopped, such as when it is effectively managing IBD symptoms, dose adjustments or the addition of treatments to manage the pulmonary side effects may be necessary. Uncontrolled cases, despite medical treatment, should warrant another systemic review of treatable traits. Identifying and treating undiagnosed factors such as silent aspirations, GERD, and environmental exposures could significantly improve respiratory symptoms and prevent the need of additional immunosuppressive treatment [97].

The existing literature indicates that, regarding the prognosis for patients with IBD-related pulmonary complications, those with CD are at an increased risk of mortality from gastrointestinal and pulmonary causes [98]. The severity of IBD appears to be a significant prognostic factor for pulmonary complications, with patients experiencing active IBD being more likely to develop severe pulmonary complications and facing higher mortality rates [99]. Thromboembolic complications, including pulmonary embolism, are associated with high mortality in IBD patients, with one study reporting a 25% mortality rate among IBD patients who experienced thromboembolic events [55,100]. Data regarding long-term outcomes of pulmonary manifestations of IBD are scarce and should be the focus of future research.

## 7. Future Directions for Research on Pulmonary Manifestations of IBD

The exploration of pulmonary manifestations in IBD is an evolving field. While significant strides have been made, there remain several gaps in the literature that warrant further investigation. Large-scale epidemiological data and appropriate screening tools, an emphasis on drug-related pulmonary side effects, prospective controlled studies to evaluate therapy effects, and guidelines for correct multidisciplinary management are some of the gaps that currently exist (Table 3). Targeted research addressing these gaps will enhance the understanding of pulmonary complications of IBD, earlier diagnosis, and better treatment, ultimately enhancing patient care and outcomes.

## 8. Conclusions

This literature review has synthesized the current body of knowledge regarding the pulmonary manifestations of IBD, highlighting the clinical presentations, diagnostic challenges, underlying pathophysiology, associations with other chronic diseases, management strategies, and the implications of drug-induced complications. Through this comprehensive analysis, it is clear that pulmonary involvement in IBD is an underdiagnosed and important extra-intestinal manifestation that can significantly affect patient outcomes and quality of life.

As such, it is crucial to maintain a high index of suspicion of lung disease when treating patients with IBD, so that timely and effective treatment can be initiated, preventing complications.

### Top of Form

The complexity of these manifestations requires a multidisciplinary strategy that integrates the expertise of gastroenterologists, pulmonologists, radiologists, and other specialists to optimize patient care.

There are multiple gaps in the current literature, particularly in the areas of epidemiology, pathophysiology, long-term impacts of treatments, and the development of standardized care pathways. These gaps present opportunities for future research, which is essential for deepening our understanding of the interplay between IBD and pulmonary health.

## Figures and Tables

**Table 1:** Description of pulmonary manifestations linked to various drugs commonly used in the treatment of IBD.

Type of Drug	Potential Pulmonary Adverse Events
5-Aminosalicylic Acid (5-ASA) and Sulfasalazine	ILDs, hypersensitivity pneumonitis, eosinophilic pneumonia, or nonspecific interstitial pneumonitis [42,43]
Methotrexate	Acute or chronic pulmonary toxicity, manifesting as cough and dyspnea [44,45]
Advanced therapies	- Anti-TNF therapy—respiratory tract infections and pneumonia, reactivation of latent tuberculosis, and, rarely, drug-induced lupus [46,47]. - Anti-integrin therapies—drug-induced pneumonitis [48] - Janus kinase (JAK) inhibitor—increased risk of infections and prothrombotic events [49].
Corticosteroids	Long-term use can lead to opportunistic infections [50]

Abbreviations: ILD, interstitial lung disease.

**Table 2:** Overview of the diagnostic approaches and potential findings in assessing pulmonary complications associated with IBD.

Diagnostic Tool	Findings
Pulmonary Function Tests	Obstructive or restrictive patterns. Decreased diffusion capacity in interstitial lung disease. Might be normal in mild/early disease [73]
Chest X-ray	Patterns of interstitial lung disease, airway dilation, pleural involvement. Might be non-specific.
High-resolution computed tomography	More sensitive than X-ray, can identify features such as bronchial wall thickening, airway dilation, and mucoid impaction that are indicative of bronchiectasis, tree-in-bud patterns, ground-glass opacities, or honeycombing indicative of fibrotic changes [74,75].
Bronchoscopy	Detection of causative pathogens by bronchial washings. Bronchoalveolar lavage for cell analysis to identify lymphocytosis, eosinophilia, and CD4:CD8 ratio.
Lung Biopsy	Can provide definitive histological evidence of pulmonary involvement, revealing patterns of inflammation, fibrosis, or granuloma formation.

**Table 3:** Description of gaps in the literature and suggestions of specific actions that need to be taken.

Aspect	Research Needs
Epidemiological Data	Conduct large-scale, longitudinal studies to better understand the prevalence, incidence, and burden of pulmonary manifestations in IBD.
Pathophysiological Mechanisms	Elucidate underlying mechanisms linking IBD and pulmonary manifestations, focusing on the gut–lung axis, immune response, genetic predisposition, and environmental factors.
Impact of Biological Therapies	Investigate the long-term pulmonary health impact of biologic therapies through post-marketing surveillance studies and registry data to understand respiratory side effects.
Diagnostic and Screening Tools	Develop and validate screening tools and biomarkers for the early detection of pulmonary involvement in IBD, with high sensitivity and specificity, to enable earlier interventions and potentially improve outcomes. Standardized protocols for assessing pulmonary involvement in IBD can enhance the reliability and efficacy of the diagnosis.
Treatment Efficacy and Safety	Conduct randomized controlled trials to assess the efficacy and safety of treatments for pulmonary manifestations in IBD, focusing on newer pharmacological agents and other treatment modalities.
Multidisciplinary Management Approaches	Study multidisciplinary management strategies to develop standardized care pathways for IBD patients with pulmonary manifestations.
Quality of Life Assessments	Perform studies that assess the impact of pulmonary manifestations on the quality of life of IBD patients, including patient-reported outcomes.
Long-term outcomes	Conduct comprehensive research to gather data on the long-term outcomes of pulmonary manifestations in IBD, identifying more prognostic factors and the impact on overall survival and quality of life.
Genetic and Environmental Interactions	Research genetic factors predisposing IBD patients to pulmonary manifestations and the role of environmental exposures for risk stratification and personalized medicine approaches.
